# Pharmacokinetic/pharmacodynamic investigation of raltegravir with or without lamivudine in the context of HIV-1 pre-exposure prophylaxis (PrEP)

**DOI:** 10.1093/jac/dkab136

**Published:** 2021-05-14

**Authors:** Carolina Herrera, Julianne Lwanga, Ming Lee, Suna Mantori, Alieu Amara, Laura Else, Sujan Dilly Penchala, Deirdre Egan, Elizabeth Challenger, Laura Dickinson, Marta Boffito, Robin Shattock, Saye Khoo, Julie Fox

**Affiliations:** 1 Department of Medicine, Imperial College London, London, UK; 2 Guys and St Thomas' NHS Foundation Trust and King’s College London, London, UK; 3 Department of Pharmacology, University of Liverpool, Liverpool, UK; 4 Chelsea and Westminster Hospital NHS Foundation Trust, London, UK

## Abstract

**Background:**

To characterize their potential use in pre-exposure prophylaxis (PrEP) we compared the pharmacokinetics of raltegravir and lamivudine in genital tissue against *ex vivo* tissue infection with HIV-1.

**Methods:**

Open-label trial of 36 HIV-negative females and males randomized to 7 days raltegravir 400 mg twice daily and 7 days raltegravir 400 mg+lamivudine 150 mg twice daily (after washout), or *vice versa*. Blood, saliva, rectal fluid, rectal tissue, vaginal fluid and vaginal tissue were sampled at baseline and on and off PrEP during a total of 12 days, for pharmacokinetics and antiviral activity via *ex vivo* HIV-1_BaL_ challenge. *Ex vivo* infectivity was compared with baseline. The trial has been registered in https://clinicaltrials.gov/ with the identifier NCT03205566.

**Results:**

Steady state for both drugs was reached by day 4. Dosing with raltegravir alone provided modest *ex vivo* HIV protection with higher drug levels in rectal tissue and vaginal tissue than in plasma on and off PrEP. Off PrEP, plasma and vaginal concentrations declined rapidly, while persisting in the rectum. On PrEP, the highest lamivudine concentrations were in the rectum, followed by vaginal tissue then plasma. Lamivudine washout was rapid in plasma, while persisting in the rectum and vagina. Raltegravir/lamivudine increased *ex vivo* protection on and off PrEP compared with raltegravir alone, reaching maximum protection at day 2 in rectal tissue and at day 8 in vaginal tissue.

**Conclusions:**

Raltegravir 400 mg+lamivudine 150 mg showed high levels of *ex vivo* HIV protection, associated with high drug concentrations persisting after discontinuation in vaginal and rectal compartments, supporting further investigation of these agents for PrEP.

## Introduction

Oral pre-exposure prophylaxis (PrEP) is a rapidly emerging prevention strategy that could help reduce HIV incidence globally.^1–4^ The use of daily and on-demand oral Truvada (tenofovir disoproxil fumarate/emtricitabine), a combination of two NRTIs, for PrEP has demonstrated high efficacy.^1–3^ However, its use may be limited by side effects, renal/bone toxicity and emerging drug resistance globally.^2^^,^^5^^,^^6^ Furthermore, the use of PrEP agents acting at different stages of the viral replication cycle may allow a more forgiving dosing strategy than a regimen consisting of two NRTIs.

Integrase inhibitors act late in the replication cycle and their role in PrEP is being explored as long-acting injectables.^7^ Raltegravir is well tolerated, has few drug–drug interactions^8^ and is available as a generic drug in some countries. Humanized mice models have shown that oral dosing with raltegravir prevents HIV-1 vaginal transmission^9^ and penetrates well in vaginal and gut tissues.^10^ In humans, raltegravir rapidly and extensively penetrates the female genital tract and colorectal tissue following oral dosing with higher plateau levels compared with plasma.^11–14^ However, it is not known whether raltegravir tissue penetration is sufficient to provide protection against vaginal or rectal transmission of HIV-1 or whether combination of raltegravir with another antiretroviral such as lamivudine, an NRTI, is required.

Drug efficacy in preventing mucosal transmission can be assessed by *ex vivo* challenge of mucosal tissue explants and has been used to evaluate PrEP strategies^15–18^*in vivo* in non-human primate studies^19–21^ and clinical trials.^22–27^ The *ex vivo* challenge approach allows evaluation of efficacy, proof of concept and insight into adherence requirements for oral PrEP agents, providing milestone data before embarking on large-scale efficacy studies, which can be used as licensing data should efficacy be shown. Despite the variety of models, consistent results can be obtained among different laboratories through protocol standardization.^28^

This proof-of-concept study was undertaken to define the pharmacokinetic (PK) and pharmacodynamic (PD) activity of raltegravir using *ex vivo* HIV-1_BaL_-treated vaginal and rectal tissue obtained from healthy men and women receiving a 7 day course of raltegravir 400 mg twice daily, administered alone or in combination with lamivudine.

## Methods

### Study design

**Figure 1.**
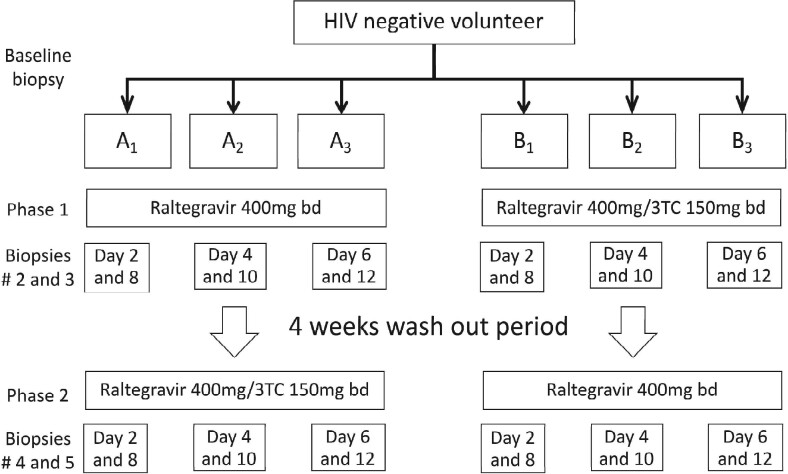
Study design. Two PrEP regimens were investigated and all 36 individuals (18 men and 18 women) received both regimens separated by a 1 month washout. Arm A started with 14 days of raltegravir 400 mg twice daily and arm B started with 14 days of raltegravir 400 mg+lamivudine 150 mg twice daily to remove sequential selection bias. Participants were randomized according to gender to one of six arms with three men and three women per block (A_1_, A_2_, A_3_, B_1_, B_2_ and B_3_). bd, twice daily; 3TC, lamivudine.

Thirty-six HIV-negative men (*n *=* *18) and women (*n *=* *18), with no sexually transmitted infections, were randomized according to gender in this open-label, PK/PD trial to one of six arms: A_1_, A_2_, A_3_, B_1_, B_2_ and B_3_ (Figure 1). The result being 6 women and 6 men per sampling arm and participants were their own controls. Arm A (A_1_, A_2_ and A_3_) received 7 days of raltegravir 400 mg twice daily, followed by a 1 month washout and then 7 days of raltegravir 400 mg/lamivudine 150 mg twice daily. Arm B (B_1_, B_2_ and B_3_) started with 7 days raltegravir 400 mg/lamivudine 150 mg twice daily, had a 1 month washout and then received 7 days of raltegravir 400 mg twice daily.

Staggered sampling was undertaken at baseline, three timepoints on PrEP and two timepoints in the 5 days after PrEP cessation according to randomization arm. Sampling took place 12 h ± 30 min after PrEP dosing. Collected samples were blood, saliva (by Salivette^®^), rectal fluid and vaginal fluid (by Weck-Cel sponges; Weck-Cel surgical spear; Medtronic Ophthalmic, Jacksonville, FL, USA), and rectal tissue and vaginal tissue (by Sarratt biopsy forceps obtaining five 3 mm × 3 mm × 1 mm biopsies). Biopsies were frozen for PK analysis or transported immediately in DMEM (median time = 30 min) to the laboratory on ice for *ex vivo* PD assays. Progesterone levels were measured on sampling visits for women.

Baseline biopsies were obtained from each individual as a control of *ex vivo* challenge. Lack of productive infection of baseline biopsies resulted in exclusion of data obtained from subsequent biopsies during the trial. Sampling from women avoided menstruation and targeted the luteal phase of the menstrual cycle.

The study was approved by the National Research Ethics Service (ref: 17/LO/0094) and registered on https://clinicaltrials.gov/ct2/show/NCT03205566?term=r-prep&cond=Hiv&rank=1. All subjects provided written informed consent.

### PK analysis

Drug concentrations in all matrices were measured by LC-MS/MS.^29^ Chromatographic separation of raltegravir and lamivudine was achieved using a Synergi polar RP column. Stable isotope-labelled internal standards (raltegravir-d6, ^15^N_2_^13^C-lamivudine and ^13^C-tenofovir diphosphate) were used for all methods. Bioanalytical method validation was carried out in accordance with FDA and EMA guidelines.^30^

#### Plasma and tissues

Drug was extracted by protein precipitation [in acetonitrile/water (5:1, v/v)]. Prior to extraction, tissues were homogenized using a MINILYS homogenizer and Precellys–Keramik kit (Bertin Technologies, Bordeaux). Calibration curves ranged between 5–5000 ng/mL (plasma) and 0.35–1000 ng/mL (tissue).

Lamivudine triphosphate (lamivudine-TP) tissue concentrations were determined using weak anion exchange chromatography. Biopsies were homogenized in a solution of methanol and 20 mM EDTA/EGTA (70:30, v/v). A biobasic AX column with a pH gradient mobile phase was used to elute lamivudine-TP and detection was performed on an SCIEX 5500 triple quadrupole mass spectrometer. The assay was validated over the concentration range of 0.075–213 pmol.

#### Saliva

Drug was extracted using solid phase extraction (SPE) [Oasis HLB (30 mg)] cartridges. The solvent phase was evaporated and reconstituted in acetonitrile/water (1:99, v/v). The calibration curve (0.5–50 ng/mL for both analytes) was prepared using saliva collected from non-medicated healthy volunteers using the Salivette^®^ method.

#### Vaginal and rectal fluids

Drug was extracted from Weck-Cel sponges with a mixture of acetonitrile/0.1% formic acid. The smples were then passed through SPE [Oasis HLB (30 mg)] cartridges as an additional sample clean-up step. The solvent phase was evaporated and reconstituted in acetonitrile/water (1:99, v/v). The volume of fluid on each sponge was predetermined by subtracting the weight of the ‘dry’ sponge prior to sample collection. The calibration curve (0.15–200 ng/sample) was constructed by spiking plasma calibration standards onto cellulose-based Weck-Cel sponges.

### PD analysis

Susceptibility to HIV infection was assessed using an *ex vivo* challenge model^15^^,^^31^ with a reference clade B R5-tropic isolate, HIV-1_BaL_,^32^ provided by the NIH AIDS Research & Reference Reagent Program (http://www.aidsreagent.org/). Vaginal and rectal biopsies were cut in explants, exposed in duplicates for 2 h to HIV-1_BaL_ at a high (10^4^ TCID_50_/mL) and a low (10^2^ TCID_50_/mL) titre and then washed with PBS. Different titres of virus were used to mimic *in vivo* transmission. Rectal explants were transferred onto gelfoam rafts (Pfizer, NY, USA) and vaginal explants onto a fresh culture plate. Explants were cultured for 15 days. Approximately 50% of the culture supernatant was harvested every 2–3 days and cultures re-fed with fresh medium in the absence of drug. Viral replication was measured as p24 concentration (INNOTEST HIV antigen mAb; Fujirebio Europe, Belgium) in culture supernatant at days 3, 7, 11 and 15.

### Statistical analysis

Drug concentrations are expressed as ng/mL of plasma or saliva. Tissue homogenate and genital/rectal fluid samples were quantified using an ng/sample calibration curve and converted into ng/mL by adjusting for recorded fluid volumes and tissue weights, assuming an equivalent density for 1 g of tissue and 1 mL of fluid.

Summary statistics of absolute drug concentrations at each visit on days 2, 4 and 6 (on PrEP) and days 8, 10 and 12 (off PrEP) are presented using the arithmetic mean and SEM. Inter-participant variability in plasma concentration following drug administration was assessed by measuring the coefficient of variation (CV = SD/mean × 100). Compartment-to-plasma ratios (parameter_COMP_/parameter_Plasma_) were derived at each visit using detectable drug levels only.

## Results

### Demographics and safety

Thirty-six subjects (18 males and 18 females) were included in the analysis (Table 1). The luteal phase could not be confirmed for all female participants (Table S1, available as Supplementary data at *JAC* Online). The study drugs were well tolerated with no serious adverse events reported.

**Table 1.** Baseline demographic characteristics; *n *=* *36

Age, median (IQR)	32 (20–50)
Gender, frequency (%)	
male	18 (50)
female	18 (50)
Ethnicity, frequency (%)	
white	23 (64)
black African	11 (30)
other	2 (6)
Weight (kg), mean ± SD	72.74 ± 13.8
BMI (kg/m^2^), mean ± SD	24.5 ± 3.60

Baseline characteristics are summarized as the mean ± SD (continuous normally distributed variables), median (IQR) (non-normally distributed variables) and frequency (%) (categorical variables).

### PK profile of raltegravir with or without lamivudine

**Figure 2.**
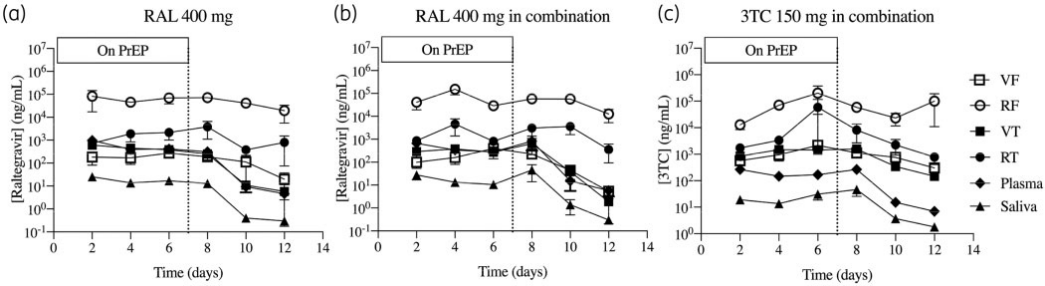
Longitudinal PK analysis. Raltegravir (a and b) and lamivudine (c) levels were measured in vaginal fluid, rectal fluid, vaginal tissue, rectal tissue, plasma and saliva at each sampling point during and after PrEP dosing with raltegravir 400 mg (a) and raltegravir 400 mg+lamivudine 150 mg (b and c). Data are mean ± SEM. The dotted line indicates the timepoint when PrEP dosing stopped. RAL, raltegravir; 3TC, lamivudine; VF, vaginal fluid; RF, rectal fluid; VT, vaginal tissue; RT; rectal tissue.

Raltegravir and lamivudine were detectable at day 2 of PrEP and concentrations reached steady state within 4 days of oral dosing in all compartments: plasma, saliva, vaginal tissue, vaginal fluid, rectal tissue and rectal fluid (Figure 2 and Table S2).

The PK profile of raltegravir was not significantly affected by lamivudine when used in combination. Raltegravir was rapidly absorbed into vaginal and rectal tissues. On day 2 of PrEP, raltegravir plasma concentrations exceeded those in vaginal fluid and vaginal tissue, suggesting a slight lag in raltegravir accumulation in the female genital tract. Higher levels of raltegravir were observed in the rectum than the female genital tract and plasma. Raltegravir tissue-to-plasma ratios on PrEP were, on average, 0.9 for vaginal tissue and 13 for rectal tissue. Off PrEP, the ratios increased to 2.0 (vaginal tissue) and 128 (rectal tissue). Raltegravir levels in rectal fluid were significantly higher than rectal tissue during and after PrEP; however, in the vagina the opposite was observed, with greater concentrations in tissue than in secretions.

Raltegravir concentrations during PrEP in plasma, rectal and vaginal samples were greater than the protein-adjusted IC_95_ for WT virus (16 ng/mL)0.8 After PrEP cessation, 86% and 58% of rectal tissue samples and 50% and 7% of vaginal tissue samples remained above the IC_95_ at days 10 and 12, i.e. days 3 and 5 post-ART, respectively, in both arms.

Rectal concentrations of lamivudine (Figure 2c) were higher on average than vaginal and plasma concentrations. Lamivudine tissue-to-plasma ratios were, on average, 8.0 for vaginal tissue and 129 for rectal tissue on PrEP and 17 (vaginal tissue) and 91 (rectal tissue) off PrEP. Furthermore, lamivudine levels in the vaginal and rectal compartments remained high after stopping PrEP, with 100% and 46% of rectal tissue samples and 60% and 43% of vaginal tissue samples above the *in vitro* IC_95_ (183.4 ng/mL)^33^ at days 10 and 12, i.e. days 3 and 5 post-ART, respectively.

Average lamivudine-TP levels in vaginal tissue were 399 pmol/g on PrEP and 305 pmol/g off PrEP (Table S3). Metabolite concentrations persisted in vaginal tissue with 71% of vaginal tissue samples detectable at day 12, i.e. day 5 post-ART. Lamivudine-TP was undetectable in all rectal tissue samples.

Significant positive correlations were observed between raltegravir and lamivudine concentrations in vaginal tissue and rectal tissue with corresponding levels in plasma (Figure S1) and mucosal secretions (Figure S2). In vaginal tissue, there was a significant log relationship between lamivudine and lamivudine-TP concentrations (*P *=* *0.0171) (Figure S3a).

Saliva and plasma concentrations of raltegravir and lamivudine were significantly correlated (r^2^>0.7; *P *<* *0.0001) (Figure S1). However, levels of raltegravir and lamivudine were lower in saliva, accounting for approximately 4% (raltegravir) and 13% (lamivudine) of total drug concentrations in plasma.

Sampling women rectally and vaginally simultaneously uniquely allowed comparison of compartments following oral dosing. During oral dosing with raltegravir (both alone and in combination with lamivudine), raltegravir levels in female rectal tissue were, on average, 3.6 ± 0.4-fold higher than in vaginal tissue and 98.1 ± 24.8-fold higher in rectal fluid than in vaginal fluid at day 6. Off PrEP, raltegravir levels remained high in the rectum, but began to decline in the vaginal compartment, resulting in 76-fold higher concentrations in rectal tissue than in vaginal tissue and 815-fold higher concentrations in rectal fluid than in vaginal fluid at day 10, i.e. day 3 post-ART (Figure S4). Similarly, lamivudine levels were consistently higher in the rectum than in the vaginal compartment of female subjects both on and off PrEP. Both raltegravir and lamivudine concentrations in vaginal tissue significantly correlated with levels in rectal tissue. For secretions, however, raltegravir vaginal fluid significantly correlated with rectal fluid, but lamivudine did not (Figure S4).

No significant gender differences were observed when comparing the raltegravir and lamivudine plasma, saliva and rectal concentrations (data not shown).

### Efficacy of raltegravir and the raltegravir/lamivudine combination against rectal and vaginal transmission

**Figure 3.**
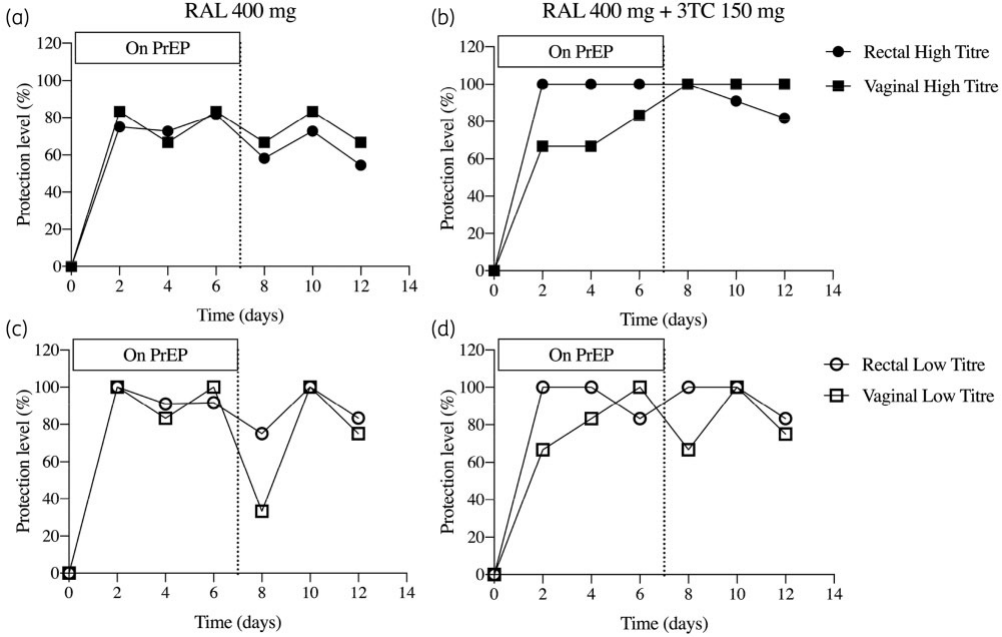
Longitudinal analysis of protection level. *Ex vivo* protection of rectal and vaginal explants was defined as day 15 p24 level >60% lower compared with day 15 p24 of baseline explants following challenge with HIV-1_BaL_ at a high titre (10^4^ TCID_50_/mL) (a and b) or a low titre (10^2^ TCID_50_/mL) (c and d). Data are the percentage of samples considered protected under this criterion at each timepoint on and off PrEP with raltegravir 400 mg (a and c) and raltegravir 400 mg+lamivudine 150 mg (b and d). The dotted line indicates the timepoint when PrEP dosing stopped. RAL, raltegravir; 3TC, lamivudine.

We evaluated the efficacy of oral PrEP in mucosal explants against challenge with HIV-1_BaL_ at two titres: a high titre routinely used to obtain productive infection of explants and a low titre to mimic *in vivo* transmission. For both titres, reductions in p24 levels compared with those measured at baseline were observed in rectal tissue and vaginal tissue after 2 days of oral dosing with raltegravir alone and progressively decreased after PrEP cessation (Figure S5). Dosing with the raltegravir/lamivudine combination resulted in a greater reduction of p24 levels in both tissues (Figure S5). We then used these reductions in p24 to define *ex vivo* protection as the percentage of cultures where infectivity after *ex vivo* challenge was reduced above a certain cut-off compared with baseline samples. We explored a range of cut-offs between 60% and 90% (Figure 3 and Figure S6). For both dosing regimens, with 60%, 70% or 80% cut-offs there was protection of rectal tissue and vaginal tissue from day 2 on drug until 5 days post-drug cessation against high and low titres. Using a 90% cut-off there was rectal, but not vaginal, protection against high and low titre challenge across all timepoints from day 2 on drug to day 5 post-drug cessation.

### PK/PD correlation

**Figure 4.**
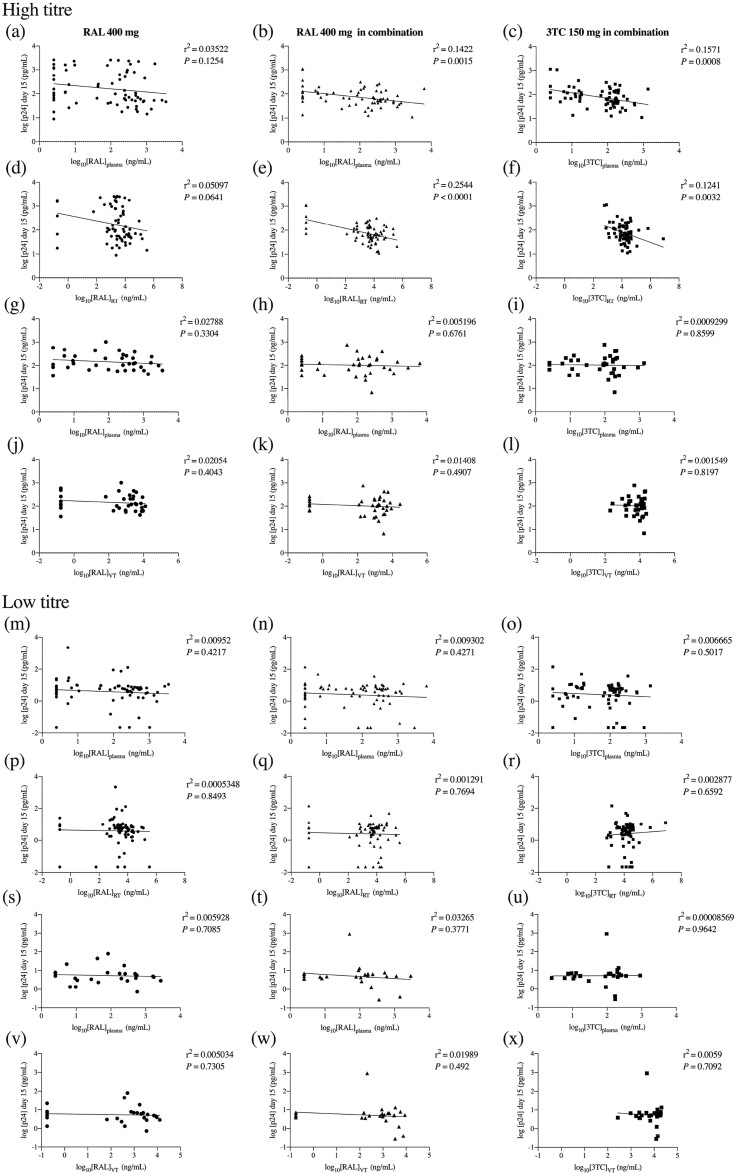
Correlations of drug concentrations with p24 levels in culture supernatants. Log-transformed p24 levels in day 15 culture supernatants of rectal (a, b, c, d, e, f, m, n, o, p, q and r) and vaginal (g, h, i, j, k, l, s, t, u, v, w and x) explants challenged *ex* vivo with HIV-1_BaL_ at a high titre (10^4^ TCID_50_/mL) or a low titre (10^2^ TCID_50_/mL) were correlated with log-transformed raltegravir and lamivudine concentrations in plasma and mucosal tissue (rectal tissue or vaginal tissue) by Pearson correlation. *P *<* *0.05 was considered statistically significant. RAL, raltegravir; 3TC, lamivudine; RT, rectal tissue; VT, vaginal tissue.

Statistically significant inverse correlations between *ex vivo* infectivity levels and drug concentrations were only observed in participants dosed with raltegravir 400 mg+lamivudine 150 mg between rectal tissue challenged with high viral titre and raltegravir and lamivudine concentrations in plasma (Figure 4b and c) and rectal tissue (Figure 4e and f). No significant PK/PD correlations were observed with low viral titre challenge infectivity data (Figure 4) or with lamivudine-TP levels in vaginal tissue (Figure S3b and c).

## Discussion

We showed that a 7 day course of raltegravir/lamivudine or raltegravir alone results in high drug concentrations at multiple HIV transmission sites by day 2 of dosing. Raltegravir concentrations were consistently higher in the rectum than plasma, saliva and the female genital tract. Higher levels of raltegravir in rectal fluid than in vaginal fluid have also been reported in animal models and in humans.^12^^,^^34^ Raltegravir persisted longest in rectal tissue and rectal fluid in parallel with persistent *ex vivo* protection and resulting in high rectal tissue-to-plasma ratios towards the end of the sampling interval, as plasma concentrations declined. Vaginal tissue and vaginal fluid raltegravir concentrations remained, on average, above the *in vitro* IC_95_ for 3 days after dosing cessation, providing protection levels above 70% with high and low *ex vivo* challenge titres. Interestingly, drug plasma levels correlated better with those in vaginal tissue than in vaginal fluid and rectal samples. Given that both drugs are metabolized via separate pathways, not surprisingly, the raltegravir PK profile was not affected by the combined dosing with lamivudine. Concentrations of lamivudine and lamivudine-TP in the female genital compartment and in the rectal tract remained above plasma and saliva levels during and after dosing, potentially explaining the continued protection during lamivudine/raltegravir off PrEP. Our study did not include a lamivudine-alone arm, limiting the full assessment of the contribution of raltegravir and lamivudine in the increased activity of the combination compared with both drugs alone. However, our data support that the combination regimen is more ‘forgiving’ than raltegravir alone and perhaps more suitable for daily or even on-demand PrEP. Further studies evaluating the activity of raltegravir/lamivudine at short timepoints post-dosing will be required to fully confirm the potential of this drug combination for on-demand PrEP.

The high raltegravir level in rectal tissue and prolonged presence shows that it accumulated in the tissue and suggests that, following oral dosing, it was absorbed into the circulation. In addition, raltegravir may accumulate within the rectum directly as unabsorbed drug carried in faeces or excreted in a conjugated form by the bile duct and de-conjugates in the large bowel back to raltegravir.

Macaque studies had shown that the luteal phase is associated with increased risk of vaginal HIV transmission;^35^ however, in our study the luteal phase could not be confirmed for all female participants and no correlation was established with PK/PD results.

All baseline rectal explants were reproducibly infected with high and low challenge titres, except in two participants: for the first, no productive infection was observed; for the second, explants cut for the high challenge titre were too small. All baseline vaginal explants challenged with the high viral titre were infected; however, only 13 of a total of 18 explants were productively infected with the low titre at baseline. Consistent with histological and immunological differences,^36^ maximum p24 concentrations were 1 log greater in rectal than in vaginal explants.

Higher levels of raltegravir in rectal tissue compared with vaginal tissue did not correlate with higher *ex vivo* protection in rectal explants. However, during oral dosing with raltegravir 400 mg+lamivudine 150 mg, a greater protection level against *ex vivo* challenge in rectal tissue than in vaginal tissue reflected higher concentrations of raltegravir and lamivudine measured in rectal samples compared with vaginal specimens, resulting in a statistically significant negative correlation between raltegravir and lamivudine concentrations and p24 levels in rectal tissue. Hence, the raltegravir/lamivudine combination maintained protection in a broader range of tissue and for longer compared with raltegravir alone, conferring protection for 5 days after stopping PrEP. The lack of statistically significant PK/PD correlations in vaginal tissue (at high viral titres) could be due to insufficient drug levels in HIV target cells within the tissue to inhibit viral replication or to tissue-specific factors such as cellular activation level and microbiota. In contrast, drug concentrations may exceed the required levels for inhibition in tissues subjected to a low viral challenge titre, thereby preventing the establishment of a concentration–effect relationship.

Currently the *ex vivo* challenge of mucosal explants remains the only practical way to address PD in humans, aside from Phase III trials, and provides an important tool for risk reduction of late-stage failure when selecting drug strategies for large studies.

The close correlation of drug levels between saliva and plasma indicates that non-invasive saliva sampling could be used to monitor adherence for people on PrEP or HIV treatment taking raltegravir and/or lamivudine.

To the best of our knowledge, this is the first report of the protective activity of raltegravir as an oral PrEP candidate where longitudinal sampling for each participant has been conducted using samples at baseline as their own control and confirms that it is rapidly absorbed with greater accumulation in rectal tissue than in vaginal tissue. Pharmacological boosting of raltegravir with lamivudine resulted in greater and more prolonged protection against *ex vivo* challenge of rectal and vaginal tissues without a change in drug levels of raltegravir in these compartments. Hence, lamivudine continues to show promise as a PrEP agent. Therefore, the raltegravir 400 mg+lamivudine 150 mg regimen is a potential PrEP candidate for people who are unable to take tenofovir-based PrEP.

## Supplementary Material

dkab136_Supplementary_Data
