# Supplementary material for: Pharmacokinetic/pharmacodynamic investigation of raltegravir with or without lamivudine in the context of HIV-1 pre-exposure prophylaxis (PrEP)
Source: J Antimicrob Chemother. 2021 May 14;76(8):2129–36. doi: 10.1093/jac/dkab136 (PMC8325523; doi:10.1093/jac/dkab136)
Supplement: dkab136_Supplementary_Data [file dkab136_supplementary_data.doc]

**Supplementary data**

**Table S1. Progesterone levels of participants at each visit**

| **Subject** | **Visit day** | **Last menstrual period** | **Progesterone levels (nM)** | **Comments** |
| --- | --- | --- | --- | --- |
| 01-009 | 13.10.17 | 28/09/2017 | 25 |  |
|  | 24.10.17 |  | 15 |  |
|  | 30.10.17 |  | 15 |  |
|  | 01.12.17 | 09/11/20017 | 2 |  |
|  | 07.12.17 |  | 1 |  |
| 01-017 | 09.01.18 | 10/12/2017 | 1 |  |
|  | 16.01.18 |  | 1 |  |
|  | 22.01.18 |  | 1 |  |
|  | 22.02.18 | 15/12/2017 | 1 |  |
|  | 28.02.18 |  | 1 |  |
| 01-023 | 29.01.18 | 10/01/2018 | <1 |  |
|  | 22.02.18 |  | <1 |  |
|  | 28.02.18 |  | <1 |  |
|  | 06.04.18 |  |  | Progesterone not collected |
|  | 12.04.18 |  | <1 |  |
| 01-001 | 19.09.17 | 08/09/2017 | 1 |  |
|  | 06.10.17 |  | <1 |  |
|  | 11.10.17 |  | <1 |  |
|  | 17.11.17 | 08/09/2017 | 1 |  |
|  | 23.11.17 |  | <1 |  |
| 01-027 | 22.01.18 | 26/12/2017 | 1 |  |
|  | 06.02.18 |  | 1 |  |
|  | 12.02.18 |  | 1 |  |
|  | 05.04.18 | 21/03/2018 | <1 |  |
|  | 11.04.18 |  | <1 |  |
| 01-044 | 14.06.18 | 30/05/2018 |  | Progesterone not collected |
|  | 19.06.18 |  | 2 |  |
|  | 25.06.18 |  |  | Progesterone not collected |
|  | 14.09.18 | 11/09/2018 |  | Progesterone not collected |
|  | 20.09.18 |  | 15 |  |
| 01-003 | 06.10.17 | 06/04/2017 | <1 |  |
|  | 12.10.17 |  | 1 |  |
|  | 18.10.17 |  | 1 |  |
|  | 24.11.17 | 16/11/2017 | <1 |  |
|  | 30.11.17 |  | <1 |  |
| 01-005 | 27.09.17 | 15/06/2017 | 1 |  |
|  | 12.10.17 |  | 1 |  |
|  | 18.10.17 |  | <1 |  |
|  | 24.11.17 |  | <1 |  |
|  | 30.11.17 |  | <1 |  |
| 01-010 | 06.10.17 | 14/09/2017 | <1 |  |
|  | 20.10.17 |  | 2 |  |
|  | 26.10.17 |  | <1 |  |
|  | 29.11.17 | 21/09/2017 | <1 |  |
|  | 05.12.17 |  | 2 |  |
| 01-011 | 10.10.17 | 01/10/2017 | <1 |  |
|  | 17.10.17 |  | 12 |  |
|  | 23.10.17 |  | 4 |  |
|  | 23.11.17 | 09/11/2017 |  | Progesterone not collected |
|  | 29.11.17 |  | 1 |  |
| 01-014 | 02.11.17 | 10/11/2017 | <1 |  |
|  | 28.11.17 |  | 17 |  |
|  | 04.12.17 |  | <1 |  |
|  | 18.01.18 | 16/12/2017 | 1 |  |
|  | 24.01.18 |  | <1 |  |
| 01-030 | 01.02.18 |  | 1 |  |
|  | 08.02.18 |  |  | Progesterone not collected |
|  | 14.02.18 |  | <1 |  |
|  | 16.03.18 | 14/03/2018 |  | Progesterone not collected |
|  | 22.03.18 |  |  | Progesterone not collected |
| 01-016 | 08.01.18 | 25/12/2017 | 1 |  |
|  | 31.01.18 |  | 1 |  |
|  | 06.02.18 |  | <1 |  |
|  | 15.03.18 | 22/02/2018 |  | Progesterone not collected |
|  | 21.03.18 |  | <1 |  |
| 01-028 | 30.01.18 | 17/01/2018 | <1 |  |
|  | 13.02.18 |  | <1 |  |
|  | 19.02.18 |  |  | Progesterone not collected |
|  | 05.04.18 | 05/03/2018 | <1 |  |
|  | 11.04.18 |  | 2 |  |
| 01-034 | 20.03.18 | 16/02/2018 | 1 |  |
|  | 12.04.18 |  | 1 |  |
|  | 18.04.18 |  | <1 |  |
|  | 24.05.18 | 18/05/2018 |  | Progesterone not collected |
|  | 30.05.18 |  | 1 |  |
| 01-015 | 14.11.17 | 15/10/2017 | 19 |  |
|  | 28.11.17 |  | 7 |  |
|  | 04.12.17 |  | 27 |  |
|  | 16.01.18 | 09/01/2018 | 1 |  |
|  | 22.01.18 |  | 19 |  |
| 01-019 | 25.01.18 | 14/01/2018 | 1 |  |
|  | 02.02.18 |  | 1 |  |
|  | 08.02.18 |  | 1 |  |
|  | 14.03.18 | 09/03/2018 | <1 |  |
|  | 20.03.18 |  | <1 |  |
| 01-020 | 18.01.18 | 26/12/2017 | 42 |  |
|  | 02.02.18 |  | 1 |  |
|  | 08.02.18 |  | 28 |  |
|  | 15.03.18 | 19/02/2018 | 67 |  |
|  | 21.08.18 |  | 4 |  |

| **Period** | **On-PrEP** | | | | | | **Off-PrEP**^b^ | | | | | |
| --- | --- | --- | --- | --- | --- | --- | --- | --- | --- | --- | --- | --- |
| **Day** | **2** | | **4** | | **6** | | **8** | | **10** | | **12** | |
| **Regimen** | **RAL** | **RAL/3TC** | **RAL** | **RAL/3TC** | **RAL** | **RAL/3TC** | **RAL** | **RAL/3TC** | **RAL** | **RAL/3TC** | **RAL** | **RAL/3TC** |
| Plasma, ng/mL^a^ | 979.8 (306.5) | 669.9 (231.4) | 394.5 (149.0) | 337.6 (92.7) | 416.7 (119.5) | 281.6  (99.1) | 341.1 (78.2) | 828.6 (510.3) | 21.6 (8.58) | 25.9  (17.4) | 6.96 (0.83) | 16.6  (9.2) |
| Undetectable (%) | 0 | 0 | 0 | 0 | 0 | 0 | 8 | 0 | 64 | 45 | 54 | 77 |
| VF, ng/mL | 182.8 (45.2) | 98.5 (45.2) | 171.1 (83.8) | 198.7 (88.7) | 274.9 (90.2) | 396.1 (254.3) | 189.1 (59.9) | 225.8 (43.3) | 140.3 (131.4) | 54.3 (31.3) | 124.2 | 12.8 (9.90) |
| Undetectable (%) | 0 | 0 | 0 | 20 | 0 | 0 | 0 | 0 | 20 | 20 | 86 | 71 |
| VF/plasma ratio | 0.16 | 0.77 | 0.72 | 1.42 | 0.76 | 0.86 | 1.01 | 0.95 | 4.60 | 2.15 | 18.7 | - |
| VT, ng/mL | 607.6 (157.3) | 289.3 (135.9) | 444.2 (193.9) | 378.2 (67.3) | 382.9 (103.4) | 273.0 (88.4) | 249.5 (110.9) | 648.2 (432.9) | 18.0 (6.4) | 48.1  (28.9) | 19.9 (19.7) | 12.3 |
| Undetectable (%) | 0 | 0 | 0 | 0 | 0 | 0 | 0 | 0 | 40 | 20 | 71 | 86 |
| VT/plasma ratio | 0.54 | 0.74 | 1.03 | 1.58 | 0.74 | 0.83 | 0.73 | 0.97 | 0.66 | 1.75 | 5.97 | - |
| RF, ng/mL^a^ | 82011 (64726) | 41269 (21937) | 45681 (10866) | 153459 (67099) | 69803 (30833) | 28928 (9008) | 72118 (21751) | 58358 (13203) | 41063 (13558) | 57147 (20258) | 19573 (13974) | 12765 (7483) |
| Undetectable (%) | 0 | 0 | 0 | 0 | 0 | 0 | 0 | 0 | 0 | 0 | 0 | 0 |
| RF/plasma ratio | 125.8 | 162.3 | 467.7 | 720.7 | 356.7 | 112.0 | 189.1 | 480.6 | 4164 | 8757 | 5026 | 658.7 |
| RT, ng/mL^a^ | 729.4 (218.1) | 888.3  (218.4) | 1899 (1058) | 4593 (3199) | 2160 (1146) | 809.1 (349.0) | 4186 (2975) | 3096 (1731) | 377.4 (181.2) | 4931 (2626) | 1161 (1047) | 576.8 (416.0) |
| Undetectable (%) | 0 | 0 | 0 | 0 | 0 | 0 | 8 | 0 | 0 | 27 | 31 | 38 |
| RT/plasma | 1.12 | 4.65 | 30.4 | 24.5 | 15.2 | 2.37 | 7.12 | 24.9 | 37.9 | 351.5 | 299.0 | 52.4 |
| Saliva, ng/mL^a^ | 25.3 (7.36) | 27.5 (10.6) | 14.9 (4.61) | 14.7 (4.76) | 17.0 (5.01) | 10.5 (3.38) | 14.1 (3.85) | 45.5 (31.6) | 1.09 (0.38) | 3.55 (2.16) | <LLQ | 1.33 |
| Undetectable (%) | 0 | 0 | 9 | 9 | 0 | 0 | 8 | 0 | 82 | 64 | 100 | 92 |
| Saliva/plasma | 0.035 | 0.036 | 0.041 | 0.043 | 0.044 | 0.040 | 0.040 | 0.044 | 0.030 | 0.451 | - | 0.038 |

**Table S2.** RAL PK parameters in all compartments during daily RAL and 3TC or RAL dosing and post-treatment cessation

Raltegravir (RAL), lamivudine (3TC). Data presented as mean (SEM). Undetectable concentrations below the assay limit of quantification (LLQ) are excluded. ^a^ Values for males and females. ^b^ Day 8 refers to up to 1 day (24h), day 10 to up to 3 days (72 h) and day 12 to up to 5 days (120 h) post-dosing cessation. Undetectable = number of samples (%) below the assay limit of quantification (LLQ).

**Table S3.** 3TC PK parameters in all compartments during daily RAL and 3TC or RAL dosing and post-treatment cessation

| **Period** | **On-PrEP** | | | **Off-PrEP**^b^ | | |
| --- | --- | --- | --- | --- | --- | --- |
| **Day** | **2** | **4** | **6** | **8** | **10** | **12** |
| **Regimen** | **3TC/RAL** | **3TC/RAL** | **3TC/RAL** | **3TC/RAL** | **3TC/RAL** | **3TC/RAL** |
| Plasma, ng/mL^a^ | 265.1 (76.5) | 146.1 (11.0) | 169.1 (19.3) | 266.4 (101.6) | 15.1 (1.80) | 8.95 (1.72) |
| Undetectable (%) | 0 | 0 | 0 | 0 | 0 | 25 |
| VF, ng/mL | 572.7 (139.6) | 956.5 (401.7) | 2151 (754.6) | 1122 (122.3) | 756.7 (186.0) | 302.2 (70.6) |
| Undetectable (%) | 0 | 0 | 0 | 0 | 0 | 0 |
| VF/plasma ratio | 5.55 | 5.69 | 11.3 | 6.47 | 52.4 | 44.2 |
| VT, ng/mL | 843.9 (149.9) | 1490 (237.3) | 1438 (133.3) | 1558 (206.3) | 342.0 (98.7) | 147.9 (39.7) |
| Undetectable (%) | 0 | 0 | 0 | 0 | 0 | 0 |
| VT/plasma ratio | 6.42 | 10.2 | 7.42 | 7.69 | 23.8 | 21.6 |
| VT 3TC-TP, pmol/g | 210.1 (170.2) | 301.7 (84.8) | 563.2 (238.4) | 655.4 (278.5) | 107.9 (32.9) | 111.7 (14.6) |
| Undetectable (%) | 20 | 20 | 0 | 20 | 20 | 29 |
| RF, ng/mL^a^ | 12793 (4284) | 71231 (22612) | 203177 (171245) | 58866 (12206) | 23200 (11426) | 101126 (90231) |
| Undetectable (%) | 0 | 0 | 0 | 0 | 0 | 0 |
| RF/plasma ratio | 103.4 | 524.9 | 982.7 | 488.0 | 1410 | 11232 |
| RT, ng/mL^a^ | 1722 (235.1) | 3353 (833.3) | 1899 (57168)^c^ | 8118 (5428) | 2246 (1325) | 777.2 (367.7) |
| Undetectable (%) | 0 | 0 | 0 | 0 | 0 | 0 |
| RT/plasma | 10.7 | 26.6 | 13.4 | 46.4 | 142.3 | 85.2 |
| Saliva, ng/mL^a^ | 19.1 (4.77) | 13.7 (2.13) | 30.8 (12.2) | 46.0 (20.4) | 3.70 (0.47) | 1.99 (0.38) |
| Undetectable (%) | 0 | 0 | 0 | 0 | 0 | 8 |
| Saliva/plasma | 0.079 | 0.101 | 0.204 | 0.152 | 0.276 | 0.252 |

Raltegravir (RAL), lamivudine (3TC). Data presented as mean (SEM). Undetectable concentrations below the assay limit of quantification (LLQ) are excluded. ^a^ Values for males and females. ^b^ Day 8 refers to up to 1 day (24h), day 10 to up to 3 days (72 h) and day 12 to up to 5 days (120 h) post-dosing cessation. ^C^ Single sample excluded as a statistical outlier, potential faecal contamination of tissue biopsy. Undetectable = number of samples (%) below the assay limit of quantification (LLQ).

**Figure S1.** PK correlations between plasma and mucosal samples. Log transformed raltegravir (RAL) and lamivudine (3TC) levels on- and off-PrEP measured in plasma were correlated with those quantified in saliva (A, B, C), RT (D, E, F), RF (G, H, I), VT (J, K, L) and VF (M, N, O) by Pearson correlation. *P* < 0.05 was considered statistically significant.

**Figure S2.** PK correlations between mucosal tissues and secretions. Log transformed raltegravir (RAL) and lamivudine (3TC) levels on- and off-PrEP measured in RT (A, B, C) and VT (D, E, F) were correlated with those quantified in corresponding fluids (RF and VF) by Pearson correlation. *P* < 0.05 was considered statistically significant.

**Figure S3.** Correlations of pharmacological parameters for 3TC-TP in female genital tract. (A) Log transformed lamivudine (3TC) and 3TC-triphosphate (3TC-TP) levels on- and off-PrEP measured in VT were correlated by Pearson correlation. (B, C) Log transformed p24 levels in day 15 culture supernatants of vaginal explants challenged *ex* vivo with HIV-1_BaL_ at high (10^4^ TCID_50_/mL) or low titre (10^2^ TCID_50_/mL) were correlated with log transformed 3TC-TP concentrations in VT by Pearson correlation. *P* < 0.05 was considered statistically significant.

**Figure S4.** PK profile in mucosal compartments from female participants. Longitudinal raltegravir (RAL) (A, B) and lamivudine (3TC) (C) levels in secretions (VF, RF) and biopsies (VT, RT); and PK correlations in tissues (VT and RT) (D, E, F) and secretions (VF and RT) (G, H, I) collected on- and off-PrEP in female participants dosed with RAL 400 mg (A, D, G) and RAL 400 mg + 3TC 150 mg (B, C, E, F, H, I).

**Figure S5.** Longitudinal PD analysis in mucosal tissue. Rectal and vaginal biopsies obtained at different time points on- and off-PrEP were cut into explants and challenged in duplicates with HIV-1_BaL_ at a high (10^4^ TCID_50_/mL) (A, B, C) or a low titre (10^2^ TCID_50_/mL) (D, E, F) within 1 h of resection. Following 2 h of incubation, explants were washed with PBS and cultured for 15 days. The concentrations of p24 in culture supernatants from rectal (R p24) and vaginal explants (V p24) harvested at day 15 were quantified by ELISA. Data are mean (± SEM). Dotted line indicates time point when PrEP dosing stopped.

**Figure S6.** Longitudinal analysis of protection level with different cut-off for protection. *Ex vivo* protection of rectal and vaginal explants was defined as day 15 p24 level > 70% , > 80% or > 90% lower compared to day 15 p24 of baseline explants following challenge of rectal (●, ○) and vaginal explants (■, □) with HIV-1_BaL_ at a high (10^4^ TCID_50_/mL) (solid symbols) (A-F) or a low viral titre (10^2^ TCID_50_/mL) (open symbols) (G-L). Data are % of samples considered protected under this criterion at each time point on- and off-PrEP with raltegravir (RAL) 400 mg (A-C, G-I) and RAL 400 mg + lamivudine (3TC) 150 mg (D-F, J-L). Dotted line indicates time point when PrEP dosing stopped.

**

**

**
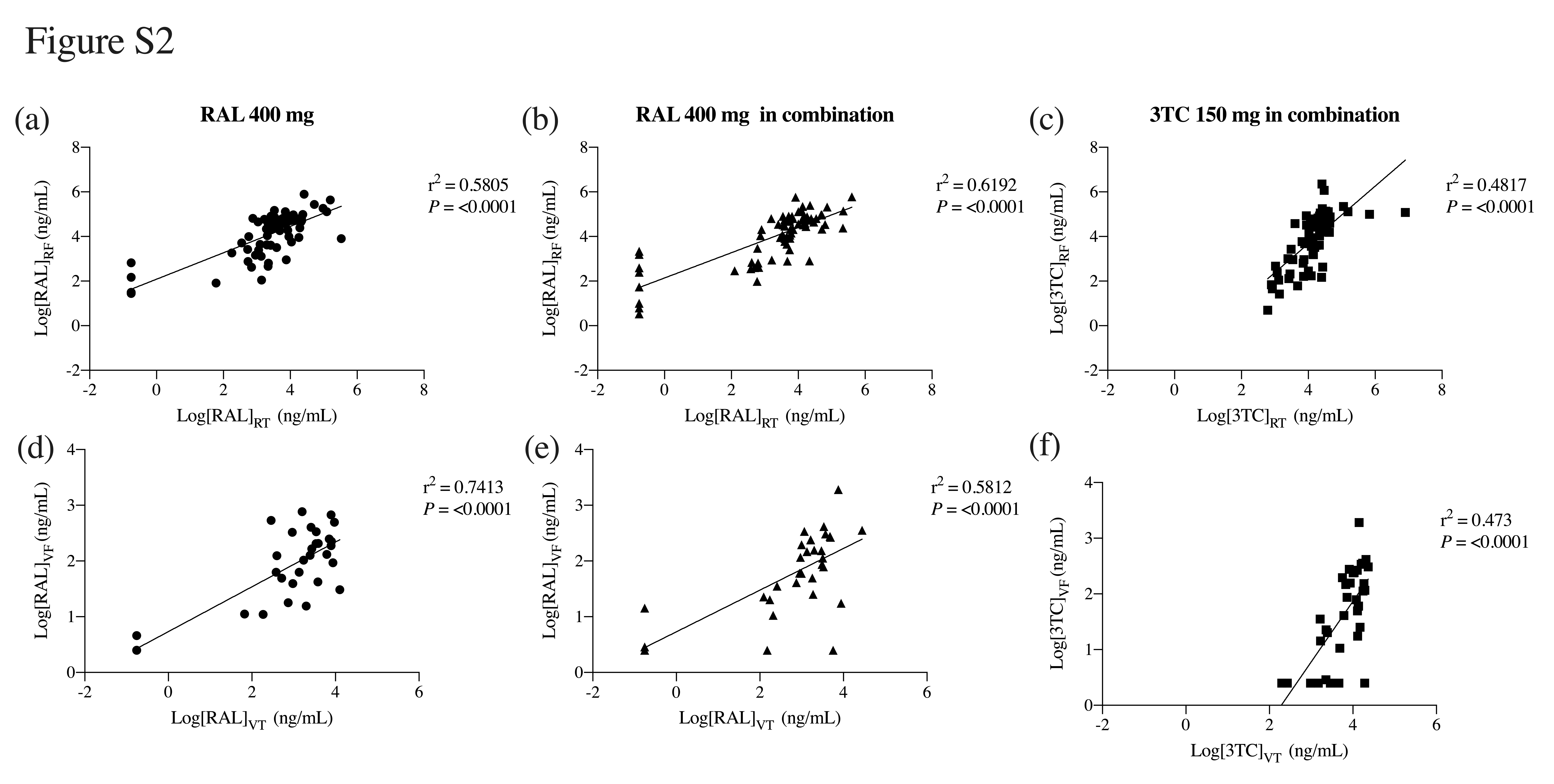
**

**
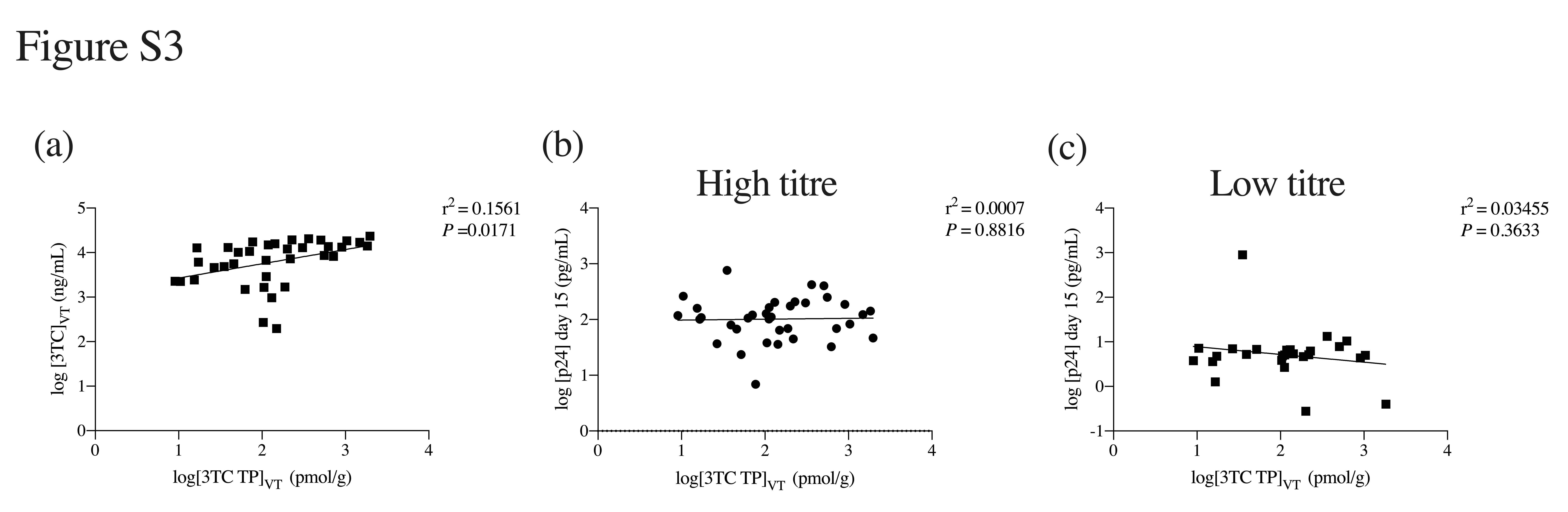
**

**
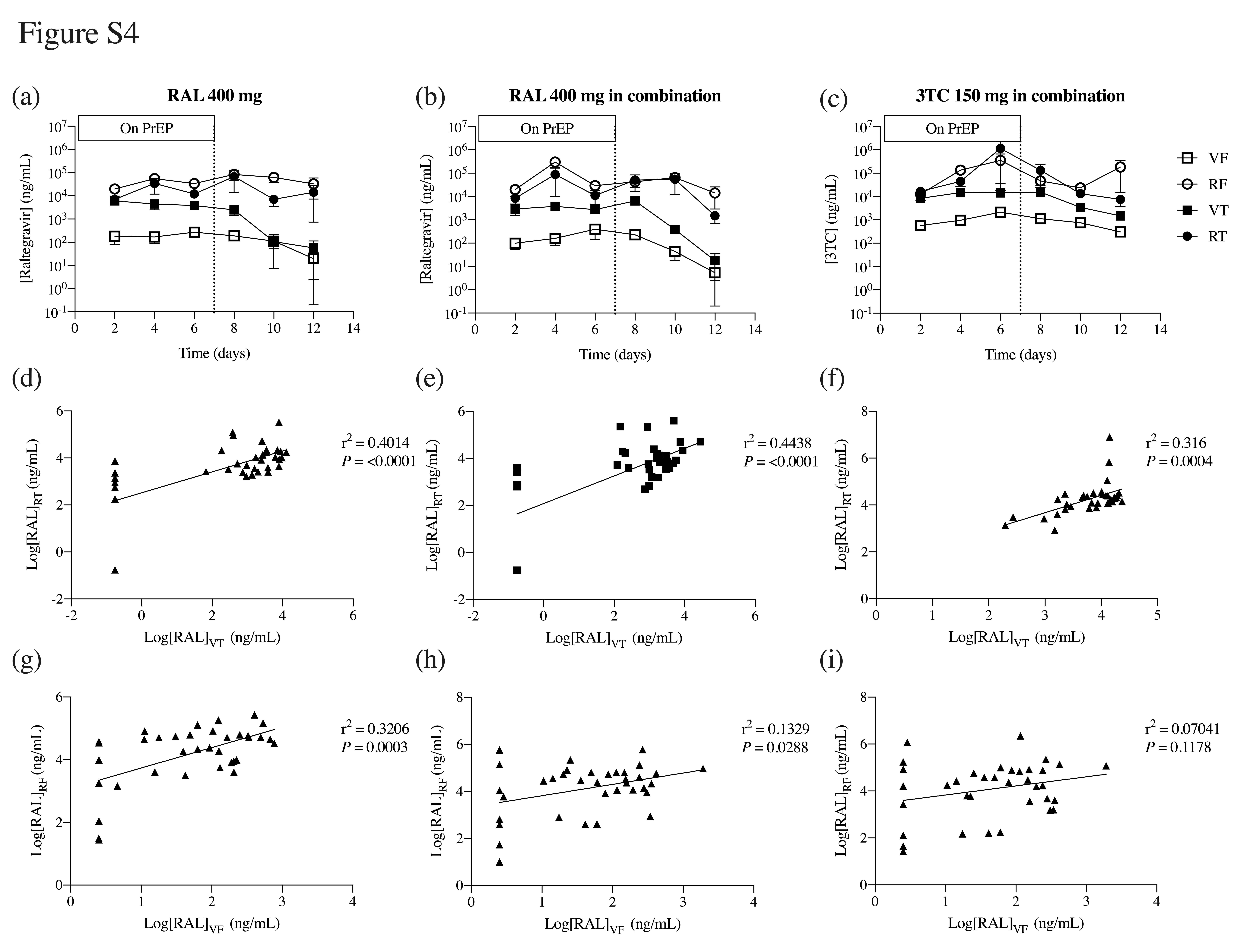
**

**
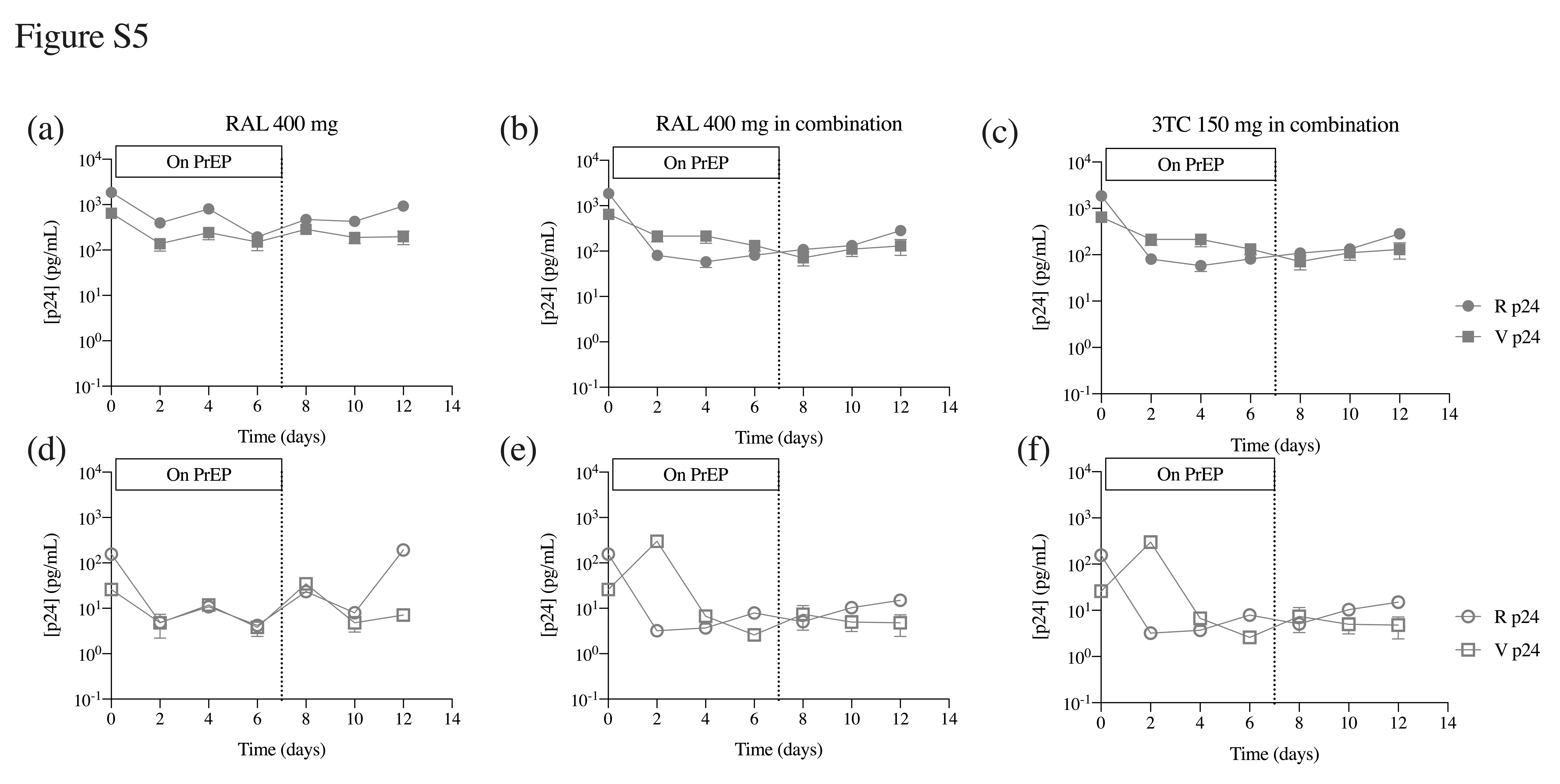
**

**

**
